# SEPT9_i1 and Septin Dynamics in Oncogenesis and Cancer Treatment

**DOI:** 10.3390/biom14091194

**Published:** 2024-09-22

**Authors:** Piotr Jędrzejczak, Kamil Saramowicz, Justyna Kuś, Julia Barczuk, Wioletta Rozpędek-Kamińska, Natalia Siwecka, Grzegorz Galita, Wojciech Wiese, Ireneusz Majsterek

**Affiliations:** Department of Clinical Chemistry and Biochemistry, Medical University of Lodz, 90-419 Lodz, Poland; piotr.jedrzejczak@stud.umed.lodz.pl (P.J.); kamil.saramowicz@stud.umed.lodz.pl (K.S.); justyna.kus1@stud.umed.lodz.pl (J.K.); julia.barczuk@stud.umed.lodz.pl (J.B.); wioletta.rozpedek@umed.lodz.pl (W.R.-K.); natalia.siwecka@stud.umed.lodz.pl (N.S.); grzegorz.galita@umed.lodz.pl (G.G.); wojciech.wiese@stud.umed.lodz.pl (W.W.)

**Keywords:** SEPT9_i1, septins, carcinogenesis, cytoskeleton, metastasis, chemoresistance, forchlorfenuron, septin inhibition

## Abstract

Despite significant advancements in the field of oncology, cancers still pose one of the greatest challenges of modern healthcare. Given the cytoskeleton’s pivotal role in regulating mechanisms critical to cancer development, further studies of the cytoskeletal elements could yield new practical applications. Septins represent a group of relatively well-conserved GTP-binding proteins that constitute the fourth component of the cytoskeleton. Septin 9 (SEPT9) has been linked to a diverse spectrum of malignancies and appears to be the most notable septin member in that category. SEPT9 constitutes a biomarker of colorectal cancer (CRC) and has been positively correlated with a high clinical stage in breast cancer, cervical cancer, and head and neck squamous cell carcinoma. SEPT9_i1 represents the most extensively studied isoform of SEPT9, which substantially contributes to carcinogenesis, metastasis, and treatment resistance. Nevertheless, the mechanistic basis of SEPT9_i1 oncogenicity remains to be fully elucidated. In this review, we highlight SEPT9’s and SEPT9_i1’s structures and interactions with Hypoxia Inducible Factor α (HIF-1 α) and C-Jun N-Terminal Kinase (JNK), as well as discuss SEPT9_i1’s contribution to aneuploidy, cell invasiveness, and taxane resistance—key phenomena in the progression of malignancies. Finally, we emphasize forchlorfenuron and other septin inhibitors as potential chemotherapeutics and migrastatics.

## 1. Introduction

Cancer is one of the leading causes of death worldwide and is among the most significant issues that modern healthcare systems face [1]. Despite advancements in surgery, chemo-, radiotherapy, and targeted therapy, which vastly improve patients’ survivability and quality of life, cancer mortality remains high [2]. Recent discoveries shed light on the role of underappreciated cellular mechanisms, including cytoskeletal dynamics and cellular transport [3,4]. Given the pivotal role of the cytoskeleton in regulating cell shape, motility, division, and intracellular signaling pathways, cytoskeletal research holds paramount importance in the field of oncology. Aberrations in cytoskeletal dynamics are frequent in cancer cells, contributing to their invasive behavior and ability to disseminate throughout the body [5,6]. Septins, integral components of the cytoskeleton, have been increasingly recognized for their involvement in tumor development, progression, and metastasis [7,8].

Septins represent a group of relatively well-conserved GTP-binding proteins capable of forming complex cytoplasmic structures [9], discovered over 50 years ago in yeast [10]. They were initially linked to yeast bud neck formation and analogous processes in eukaryotic cells, including the formation of the cleavage furrow in mammalian cells [11,12]. In the human genome, there are thirteen septin genes, which produce various isoforms. These isoforms combine in different ways to form diversified palindromic hetero-oligomers and higher-order structures [13]. Septins have been implicated in multiple cellular processes, including membrane dynamics [14], cellular transport [15], and regulation of actin and microtubule dynamics [16]. Numerous septin isoforms have been associated with various diseases, with a particular emphasis on malignancies and neurodegenerative diseases [17]. Septin 9 (SEPT9) is associated with a diverse spectrum of neoplasms—abnormal and excessive tissue growth, which covers benign neoplasms and malignant tumors (cancers). SEPT9, while being a key component of larger septin complexes, appears to play an essential role in driving tumor formation (neoplasia) by interacting with numerous pro-oncogenic factors. SEPT9 was initially identified in treatment-related acute myeloid leukemia as a fusion partner of the histone methyltransferase Mixed-Lineage Leukemia (MLL) and hence named MLL Septin-like Fusion (MSF) [18]. However, SEPT9’s role has since been extended to many other neoplasms [19]. Most notably, it serves as a biomarker of colorectal cancer (CRC) [20]. Examination of free circulating DNA to assess the methylation of specific CpG islands within the SEPT9 gene was the first blood-based tool for CRC screening approved by the FDA [20]. Such use of SEPT9 methylation as a screening tool has also been proposed for other malignancies, including liver, breast, prostate, lung, and gastric cancer [21,22,23,24,25,26]. High expression of SEPT9 has also been positively correlated with a high clinical stage in breast cancer, cervical cancer, and head and neck squamous cell carcinoma. Furthermore, SEPT9 expression has been linked to poor clinical outcomes and resistance to treatment [27,28,29,30].

The most extensively researched isoform of SEPT9 is SEPT9_i1, which is closely associated with oncogenesis and is involved in various cellular processes that facilitate malignant transformation [31,32]. Although a substantial body of evidence supports the clinical importance of SEPT9 as a biomarker and a vital contributor to carcinogenesis, the mechanistic basis for its significance remains unclear. Hence, this review aims to provide mechanistic insights into the specific role of SEPT9_i1 in carcinogenesis and explore potential clinical applications of septin inhibitors.

## 2. Septin 9 and SEPT9_i1—Structure as Basis of Protein Interactions

In humans, there are 13 protein-coding septin genes and their products are organized into four homologous groups: SEPT2 group (SEPT1, SEPT2, SEPT4, SEPT5), SEPT3 group (SEPT3, SEPT9, and SEPT12), SEPT6 group (SEPT6, SEPT8, SEPT10, SEPT11, and SEPT14), and SEPT7 group (only SEPT7) [33]. These groups are distinguished based on their sequence similarities and structural characteristics of the C-terminus. Septin genes’ transcription products undergo alternative splicing, significantly expanding the group of septin isoforms [34]. While several septins, including SEPT2, SEPT6, SEPT7, and SEPT9, are expressed ubiquitously [35], others display tissue or cell-specific expression, as exemplified by SEPT1 expressed specifically in lymphocytes and CNS [7]. Certain septins, including SEPT9, play a key role in maintaining cell viability and promoting cellular differentiation, as their knockout is lethal at the embryonic stage [36,37]. In contrast, other septins (e.g., SEPT6) appear to be dispensable for cell survival, as their knockout does not lead to observable phenotypic changes [38,39].

In general, septins are small GTP-binding proteins with a phosphate-binding loop (P-loop) motif [40,41]. Their primary sequence consists of three main domains: a central G-domain, an N-terminal domain, and a C-terminal domain [35]. The G-domain, usually the largest, is highly conserved and responsible for GTPase activity. It contains structural motifs necessary for GTP binding, P-loop formation, and the attachment of magnesium cofactors [40]. This domain is crucial for septin oligomerization and protein interactions. The N-terminal domain, more variable among septins, includes structured and unstructured regions [9]. The structured part contributes to membrane phospholipid binding and protein interactions, while the unstructured part is responsible for septin’s variability. Lastly, the C-terminal domain features coiled-coil motifs that play a role in stabilizing septin oligomers and assembling them into filaments [42].

**Figure 1:**
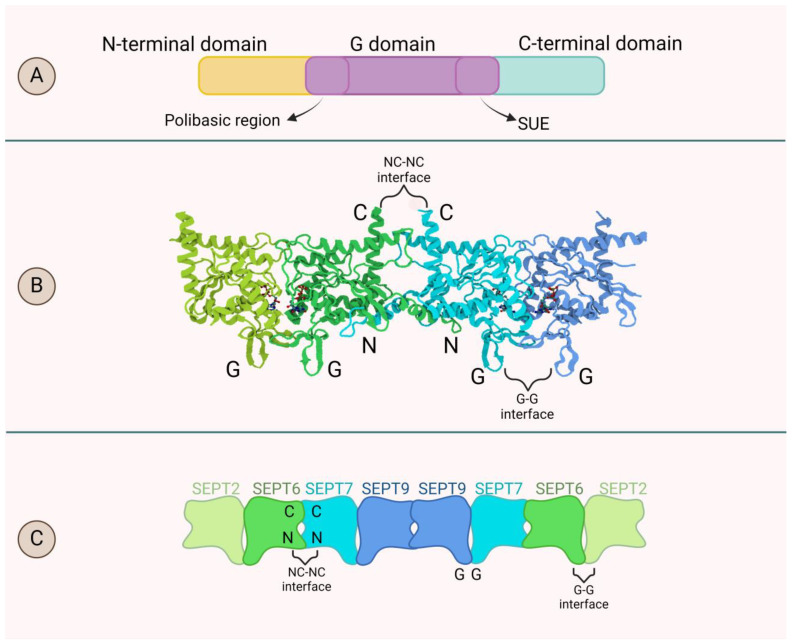
Schematic representation of septin structure: (**A**) The structure of septins is characterized by three distinct domains: the N-terminal domain, the guanine nucleotide-binding domain (also known as the G-domain), and the C-terminal domain. The largest, G-domain, is located in the center, with a highly conserved polybasic region and SUE (septin unique element) on its respective ends. (**B**) Septins’ subunits connect through their G-domains (forming G interface) as well as through their amino-terminal and carboxy-terminal regions (forming NC interface), allowing them to assemble into filaments by joining end-to-end. (**C**) Schematic representation of septin octamer. Septins from different groups assemble into complexes composed of either 6 (hexamers) or 8 (octamers) septins, with each septin present in two copies.

Septins use their domains as a binding interface to build hetero-oligomeric complexes. Monomeric septins interact with each other through the G-G interface (formed by the G domains) and the NC-NC interface (formed by the N-terminal and C-terminal domains) [43]. In mammals, endogenous septins from different groups combine to form symmetrical, predominantly palindromic hetero-hexamers or hetero-octamers, representing core particles involved in the formation of highly ordered structures. The prototypical model of the septin core particle structure is the SEPT2-SEPT6-SEPT7-SEPT7-SEPT6-SEPT2 hexamer, in which SEPT2 forms a “face-to-face” G-G interface with SEPT6, and SEPT6 forms a “back-to-back” NC-NC interface with SEPT7. SEPT9 may then fuse with this particle to form a G-G interface with SEPT7 and an NC-NC interface with another SEPT9, thereby leading to the formation of a hetero-octamer [44]. The formation of these ordered hetero-oligomers is a result of the intrinsic diversity in structure and function of different septin isoforms, including their affinity for GTP binding and hydrolysis, as well as the extrinsic involvement of other regulatory proteins and interactions with the cell membrane [13,45]. Septins undergo various posttranslational modifications, including phosphorylation, acetylation, and SUMOilation (attachment of Short Ubiquitin-like Modifier Peptide (SUMO) to the protein), which affects both heterooligomer formation and assembly into higher structures [46]. Other proteins contribute to septin assembly more directly, for example, Binder of Rho GTP-ase (BORG) proteins enhance the attachment of SEPT2 to SEPT6-SEPT7 heterodimer [47]. It was also shown that phosphatidylinositol-4,5-bisphosphate, a crucial component of cell membranes binds and promotes polymerization of septins [48]. Upon these interactions, hetero-oligomeric septin complexes can further attach end-to-end to form non-polar filaments, similar to how the intermediate filaments are formed. The septin filaments further organize themselves into more complex structures that may take on different shapes, such as rings or bundles [49]. A model of the septin primary sequence, the process of basic septin interactions, and the structure of the septin hetero-octamer are illustrated in Figure 1. 

SEPT9 features one of the most extended N-terminal domains, which, in the case of the long isoforms (i1, i2, and i3), is nearly equal in size to the G domain. The long variable N-terminal domain predisposes SEPT9 to have multiple splicing transcripts, the number of which, with the progress of bioinformatics analysis, is estimated to exceed the previously proposed 18 potential isoforms [50,51]. The unique N-terminal domain of SEPT9 isoforms was found to directly interact with other components of the cytoskeleton, including actin filaments and microtubules. Furthermore, differences among SEPT9 isoforms influence the specificity of those interactions. Notably, long isoforms of SEPT9 (i1, i2, i3) may directly interact with F-actin through the variable part of the N-terminal domain [52]. This distinguishes SEPT9 from other septins, which can only bind F-actin indirectly via interactions with adaptor proteins [16]. SEPT9 directly crosslinks actin filaments, which may be implicated in increasing epithelial cell motility [53]. In addition, SEPT9 competes for binding sites with myosin and cofilin, important drivers of actin depolymerization, thereby increasing the stability of actin filaments. SEPT9 has been shown to interact with both dynein and dynactin, key components of the cytoskeletal motor apparatus, thereby participating in the retrograde transport of lysosomes [54]. Lastly, SEPT9 has been found to maintain the structural and functional integrity of the Golgi apparatus, potentially shedding light on the role of septins in regulating membrane organelle dynamics [55]. Given that septins in nature exist in complexes, some studies based on simplified models of single septin subunits may not reflect accurately septin dynamics [52,54]. Observations of septin interactions in octamer-containing filaments and their function in human cells may therefore yield results with greater translatability [56,57].

Recently, SEPT9_i1 has been demonstrated to be the only septin isoform possessing a structural motif in the N-terminal domain that resembles the microtubule-binding domains (MBDs) of canonical human microtubule-associated proteins (MAPs) [57]. Upon direct binding to microtubules, SEPT9_i1 enhances their stability and recruits unpolymerized tubulin molecules to join the plus ends of microtubules, thereby contributing to their growth [58]. Another recent finding regarding the unique properties of the i1 isoform is the discovery of a Nuclear Localization Signal (NLS) within the unique MBD-like part of the SEPT9_i1 structure [59]. NLS is a sequence of amino acids recognized by importins and responsible for initiating the transport of certain proteins into the nucleus [60]. Here, NLS overlaps with the part responsible for microtubule binding, which strengthens the claim that minor structural differences between septins may have far-reaching consequences regarding their function.

**Figure 2:**
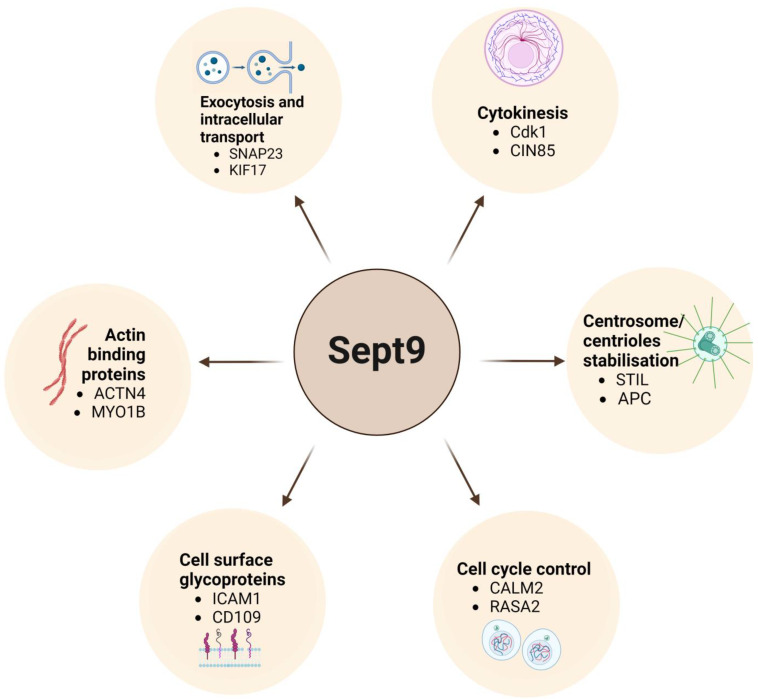
The schematic representation highlighting examples of the diverse roles of SEPT9 in essential cellular processes, such as exocytosis, cytokinesis, centrosome stabilization, cell cycle regulation, actin binding, and cell surface interactions, mediated through its specific interactions with various protein partners. Abbreviations: SNAP23—Synaptosome Associated Protein 23, KIF17—Kinesin Family Member 17, Cdk1—Cyclin-Dependent Kinase 1, CIN85—Cbl-interacting protein of 85 kDa, STIL—SCL/TAL1 Interrupting Locus, APC—Adenomatous Polyposis Coli, CALM2—Calmodulin 2, RASA2—RAS P21 Protein Activator 2, ICAM1—Intercellular Adhesion Molecule 1, CD109—Cluster of Differentiation 109, ACTN4—Alpha-Actinin 4, MYO1B—Myosin 1B.

Higher-order septin complexes can interact with other cytoskeleton elements and regulatory proteins. For example, higher-order septin complexes, such as filaments, facilitate the formation of microtubule protrusions upon actin polymerization through their interaction with BORG proteins [61]. Another study discovered that the interaction between septins, actin, and microtubules is regulated by the nucleotide status of Cdc42 and its effectors in the BORG family. After treatment with paclitaxel, a drug known for its effects on microtubule stabilization, the inactivation of Cdc42 resulted in BORG protein dissociation and proteasome-mediated degradation, leading to the relocalization of septins from actin to microtubules [62]. In addition to SEPT9’s interaction with cytoskeletal proteins, recent high-throughput methods have allowed the identification of multiple non-septin partners of SEPT9, including adhesion molecules, proteins involved in cellular transport and exocytosis, and regulators of gene expression and the cell cycle. Importantly, the wide range of SEPT9 interactions across distinct subcellular compartments and functional pathways may play a substantial role in promoting tumorigenesis [63,64,65]. Several selected specific SEPT9 interactions are depicted in Figure 2.

## 3. SEPT9_i1 Contributes to Cancer Formation, Malignancy, and Resistance to Treatment

### 3.1. SEPT9_i1 Prevents Degradation, Facilitates Transport and Scaffolds for Transcription Factors

The Hypoxia Inducible Factor (HIF) pathway represents one of the most crucial connections between cancer cells and the tumor microenvironment. This cellular system of hypoxia detection and adaptation affects cancer metabolism, motility, angiogenesis, and resistance to treatment by regulating the expression of numerous oncogenic proteins [66,67,68]. One of its most relevant effectors is vascular endothelial growth factor (VEGF), which represents a feasible therapeutic target [69]. HIF-1 is a heterodimer comprised of two subunits—α and β. The β subunit (also known as ARNT) is constitutively expressed, while the α subunit is subject to various regulatory mechanisms. Under normoxic conditions (normal cellular oxygen levels), prolyl hydroxylase (PDH) enzymes label the α subunit leading to its hydroxylation. This modification marks HIF-1α for recognition by the von Hippel–Lindau (VHL) protein complex, initiating ubiquitination and subsequent proteasomal degradation [70]. However, under hypoxic conditions, PDH enzymes become inactive, allowing the α subunit to escape degradation. The stabilized α subunit translocates to the nucleus, where it heterodimerizes with HIF-1β and binds to hypoxia response elements (HREs) in the DNA, activating the transcription of genes essential for cancer progression [71,72]. Both subunits of HIF-1 belong to the bHLH-PAS protein family. The family-defining β-Helix-Loop-Helix (bHLH) and Per and Sim (PAS) domains are crucial for facilitating heterodimer formation between the α subunit of HIF-1 and the β subunit of HIF-1, enabling binding to the HRE-DNA sequence in target genes, as well as facilitating a diverse range of protein interactions [71].

One of many mechanisms of HIF-1α regulation depends on the bHLH-PAS domain as an anchor point for two competing proteins—Receptor for Activated C Kinase 1 (RACK1) and Heat Shock Protein 90 (Hsp90) [73]. Their interplay regulates HIF-1 activity independently from oxygen conditions and the PDH/VHL pathway. The Hsp90 stabilizes HIF-1α and prevents its degradation, whereas RACK1 promotes proteasome-dependent degradation of the α subunit. It was confirmed that both Hsp90 and RACK1 connect with the same PAS-A domain and directly compete for it [73]. SEPT9_i1 was shown to influence HIF-1 activity, and one of the suggested mechanisms is its involvement in this competition [74]. It was initially revealed that SEPT9_i1 is responsible for stabilizing the HIF-1α subunit within the cell and amplifying the expression of its downstream effectors. Increased expression of HIF-1-dependent proteins requires both N-terminal 25 amino acids and the middle G domain of SEPT9_i1. This interaction was later associated with SEPT9_i1’s ability to reduce RACK1-mediated proteasomal degradation of HIF-1α, and it was subsequently confirmed that three proteins compete for binding spots in the bHLH-PAS domain of HIF-1α [74]. While both RACK1 and Hsp90 bind to the PAS-A domain, SEPT9_i1 connects with the bHLH domain via its G domain. This, however, seems sufficient for inhibiting HIF-1α proteasomal degradation. Another protein interacting with the bHLH domain of HIF-1α is ARNT. Interestingly, it does not compete for its binding spot with SEPT9_i1—in fact, more HIF heterodimers are being formed in the presence of septin, which suggests that septin serves as a scaffold in this interaction.

**Figure 3:**
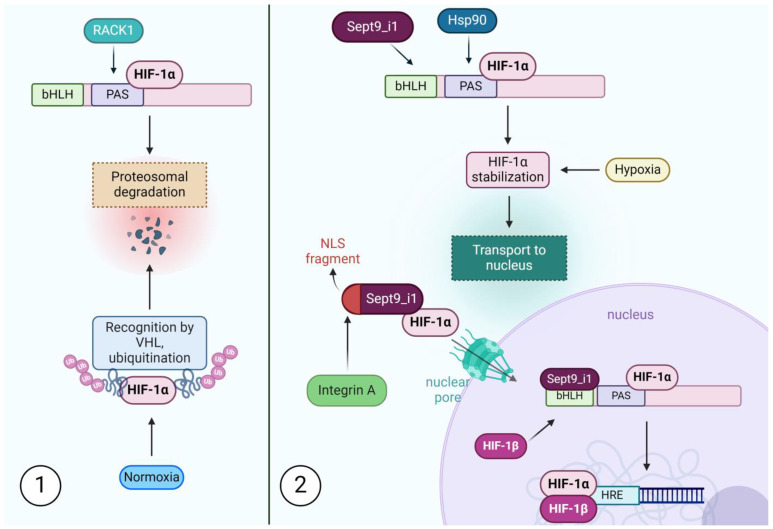
The schematic illustration of the activity of HIF1 and the role of SEPT9_i1 in regulating its stability and function: (**1**) Under normal oxygen levels, HIF1α is continuously degraded. The VHL protein recognizes and ubiquitinates HIF1α, marking it for proteasomal degradation. HIF1α contains two key regions: bHLH and PAS domains. Additionally, regardless of oxygen levels, RACK1 can promote proteasomal degradation of HIF1α by binding to its PAS domain. (**2**) Under hypoxic conditions, HIF1α is stabilized, and its degradation is inhibited. Independently of oxygen levels, HIF1α can also be stabilized through the binding of Hsp90 to its PAS domain and SEPT9_i1 to its bHLH domain. Once stabilized, HIF1α is transported to the nucleus through nuclear pores, a process facilitated by integrins. Integrins interact with the NLS part of the SEPT9_i1. Inside the nucleus, HIF1β binds to HIF1α, where SEPT9_i1 functions as an anchor. The HIF1α/β complex then binds to the Hypoxia Response Element (HRE), initiating the expression of various hypoxia-responsive genes. Abbreviations: HIF1—Hypoxia-Inducible Factor 1, VHL—von Hippel–Lindau, PAS—Per-Arnt-Sim domain, bHLH—Basic Helix–Loop–Helix domain, RACK1—Receptor for Activated C Kinase 1, Hsp90—Heat shock protein 90, NLS—Nuclear Localization Signal, HRE—Hypoxia Response Element.

However, the above-mentioned processes do not address the crucial role of the short, NLS-containing SEPT9_i1 fragment in the interaction between HIF-1α and SEPT9_i1. Like any other transcription factor, HIF requires nuclear translocation to regulate the expression of effector genes [75]. Nuclear transport of HIF-1 α is severely disrupted by introducing a hybrid protein containing N-terminal 25 amino acids from SEPT9_i1—which suggests that SEPT9_i1 amplifies HIF-1α nuclear transport in a manner dependent on this structure [59]. Nuclear transport is facilitated by a group of proteins called importins, subdivided into α and β groups. Importin α recognizes NLS sequences, binds cargo proteins, and combines with one of the β importins, facilitating the passage through the nuclear pore [76]. Due to the presence of bipartite NLS within the terminal part of SEPT9_i1, it can bind both HIF-1α and importin α, allowing for complex formation, and increasing the efficacy of nuclear shuttling. In vitro, HIF-1α connects with importins α 1, α 3, α 5, and α 7 [75]. However, in vivo, only two proteins facilitate HIF-1α transport into the nucleus—importin α 5 and α 7. SEPT9_i1 is only capable of interacting with importin α 7 [77], which suggests that there are two pathways for the nuclear import of HIF-1 α: a septin-dependent one and a septin-independent one, with the former being a potential therapeutic target through septin inhibition, especially considering its seemingly dominant role in the described interaction [77]. A schematic illustration of the interplay between septins and the HIF-1α pathway is shown in Figure 3.

Another protein that interacts with SEPT9_i1 similarly to HIF-1 α is c-Jun N-terminal kinase (JNK). JNK is a protein kinase with numerous substrates and many seemingly contradictory effects on the cell [78]. The exact role of JNK involvement in cancer development and progression is still the subject of intense research [79,80]. JNK was initially discovered as a kinase phosphorylating c-Jun, allowing it to form the Activator Protein 1 (AP1) transcription complex. AP1, among other functions, is responsible for increasing the expression of D1 cyclin, resulting in increased proliferative potential [81]. When overexpressed, SEPT9_i1 colocalizes with JNK in the nucleus and protects JNK against proteasome-dependent degradation. Accordingly, SEPT9_i1 overexpression increases cyclin D1 expression and its phosphorylation levels, supporting claims of SEPT9_i1 pro-proliferative properties.

Interestingly, while JNK’s interaction with SEPT9_i1 is facilitated through the G domain of SEPT9_i1, SEPT9_i3 was not found to have any in vitro effects on JNK activity—an observation strikingly similar to the one presented in HIF-1 studies [82]. This suggests importins’ potential involvement in this interaction, as it is described in the subsection above, which may form the basis for future research. If SEPT9_i1 is indeed involved in the nuclear shuttling of JNK via its interaction with importin α7, it may be reasonable to search for other clients of the SEPT9_i1–importin α7 complex. The above-mentioned studies support the investigations of the nuclear-shuttling pathways as well as their contribution to cancerogenesis [83] and serve as encouragement to develop SEPT9_i1 targeting drugs.

### 3.2. SEPT9_i1 Is Crucial for Integrating Septin Dynamics with Other Components of the Cytoskeleton

#### 3.2.1. SEPT9_i1 Is a Crucial Factor in Cell Invasiveness

Actin dynamics is considered essential for cancer cell motility and invasiveness. With growing evidence for SEPT9_i1’s involvement in the development of these processes, efforts have been made to determine the significance of SEPT9_i1. There are some reports suggesting SEPT9 is able to bind and bundle actin filaments and compete for binding spots on the surface of actin fibers with myosin and cofilin, proteins responsible for actin depolymerization [52,53]. Another mechanism by which SEPT9_i1 contributes to promoting cell invasiveness was recently proposed [27]. In breast cancer tissue, high SEPT9 expression was positively correlated with the expression of paxilin and nodal metastases. Further in vitro and in vivo examination showed that, indeed, vector-induced expression of SEPT9_i1 in SEPT9 knockdown cells promotes cell motility, increases the size of focal adhesions (FAs), and enables the formation of metastases in mice inoculated with tumor cells. There were several explanations proposed for this effect, several of which involved the FAK/Src/Paxilin pathway. FAK/Src/Paxilin pathway is a major pathway responsible for FA regulation. Focal Adhesion Kinase (FAK) attaches to integrins, which bind the cellular membrane with the Extracellular Matrix (ECM). Upon contact with ECM, integrins activate FAK, leading to its autophosphorylation [84]. FAK then binds Src forming a complex that recruits other structural proteins necessary for the formation of the FA complex, including paxilin. FAK-Src complex also activates further cellular signaling pathways involved in cell motility and migration [84]. SEPT9_i1 has been shown to increase FAK phosphorylation, enhancing activation of the aforementioned pathway. Furthermore, direct interaction between paxilin and SEPT9_i1 has been demonstrated in vitro and observed as colocalization in FAs. The exact mechanism of this interaction and its relationship to FAK phosphorylation remains unknown, although the authors speculate on the potential involvement of SEPT9_i1 in paxillin transport and stabilization, similar to HIF-1α. SEPT9_i1 knockdown seems to negatively affect the expression of certain proteins, including β-tubulin and β-actin. Other proteins affected similarly are Ras-related C3 botulinum toxin substrate (Rac), Ras homolog family member A (RhoA), and Cell division cycle analog 42 (Cdc42). RhoA is one of the crucial elements of the RhoA/ROCK pathway, which is considered one of the major hubs of actin dynamics, including the formation of stress fibers. RhoA upregulation may explain another effect of SEPT9_i1 overexpression—increased ROCK1 phosphorylation. Cdc42, another protein downregulated upon SEPT9_i1 knockdown, is a Rho-family GTPase, which was shown to increase FA maturation, as well as recruit SEPT9 to actin via its effector protein Binder of Rho GTP-ase 3 (BORG3), thereby promoting cell motility and invasiveness in malignant melanoma [85].

SEPT9_i1 also contributes to the formation of invadopodia—cellular structures characteristic of invasive neoplastic cells. Invadopodia represent cytoplasmatic protrusions with an actin core, which penetrate and dissolve the surrounding extracellular matrix [86]. It is speculated that nuclear deformation in cancer cells due to ECM rigidity is a crucial contributor to the formation of invadopodia, and, therefore, the formation of metastases [87]. SEPT9_i1 is crucial in maintaining the mechanical stability of the nucleus, as well as increasing the formation of juxtanuclear invadopodia by recruiting cortactin and TKS5—proteins essential for their development [88].

**Figure 4:**
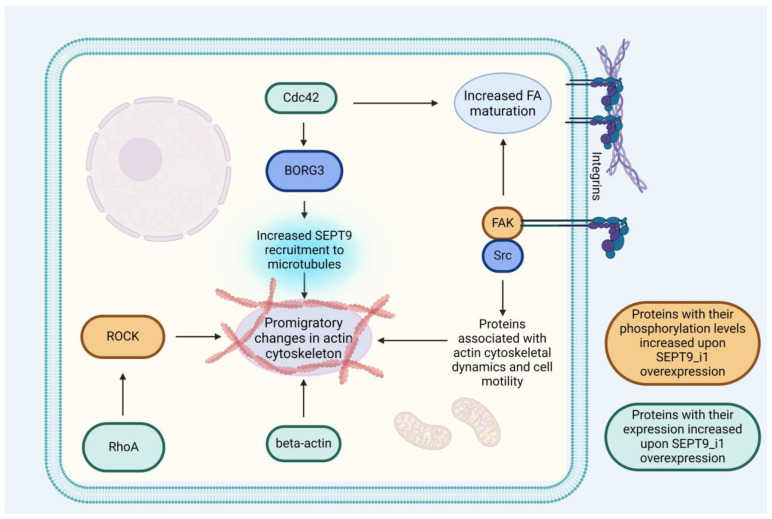
The key pathways through which SEPT9_i1 increases the metastatic capacity of cancer cells. Proteins whose expression increased after SEPT9_i1 overexpression are shown in green, while those whose phosphorylation levels increased after SEPT9_i1 overexpression are shown in orange. SEPT9_i1 overexpression increases the expression of beta-actin, a member of the family A homolog of Ras (RhoA) and an analog of cell division cycle 42 (Cdc42). Beta-actin contributes to cell motility through direct formation of actin filaments, while RhoA and Cdc42 contribute to actin cytoskeleton rearrangements through Rho-associated protein kinase (ROCK) and Binder of Rho GTP-ase 3 (BORG3), which enhance SEPT9 recruitment to and stabilization of actin filaments. Cdc42 also contributed to increased FA maturation. Another protein whose phosphorylation level increased after SEPT9_i1 overexpression was Focal Adhesion Kinase (FAK). FAK is an essential component of the Focal Adhesion complex and its function depends on phosphorylation. The FAK complex with Src also activates several pathways, additionally contributing to increased motility through actin cytoskeleton rearrangements.

Another mechanism, in which SEPT9_i1 may affect actin dynamics, involves its interaction with ARHGAP4. This Rho GTPase-activating protein is known to suppress cell motility in cancer cells and may be inhibited by SEPT9 and SEPT2 [89]. These findings further contribute to our understanding of septin’s involvement in the complex network of actin dynamics. For a graphical representation of the molecular interactions of SEPT9_i1 contributing to cancer invasiveness and metastasis, see Figure 4.

#### 3.2.2. SEPT9_i1 Interaction with Microtubules Contributes to Taxane Resistance

Taxanes are one of the essential Microtubule Targeting Agents (MTAs) used in chemotherapy. Cancer cells’ resistance to MTAs is a major clinical challenge contributing to patient mortality [90]. SEPT9_i1’s expression was found to positively correlate with a poor response to microtubule-targeting drugs, such as 2-methoxyestradiol and paclitaxel, by promoting microtubule stability and enhancing the activity of HIF-1α. [91]. Several mechanisms have been proposed to explain this correlation, including the competition for binding spots with Microtubule Associated Protein 4 (MAP4) [92]. Recently, a new mechanism explaining the SEPT9_i1 isoform’s role in the interaction with MTAs has been proposed. SEPT9_i1 is the predominant long isoform of SEPT9 in taxane-resistant cells and is essential for anchoring septin octamers to microtubules, as replacing the i1 isoform with the i3 isoform abolishes the interaction between septin octamers and microtubules [93]. Septin octamers increase the recruitment of tubulin polyglutamylation enzymes TTLL5 and TTLL11 (Tubulin Tyrosine Ligase Like 5 and 11), as well as CCP1, responsible for trimming the glutamate chains. This establishes septins as a critical regulator in that process. Interestingly, septin recruitment to microtubules seems to depend on their polyglutamylation and tubulin detyrosination/retyrosination—modifications involving the removal of the C-terminal tyrosine. This is facilitated by Tubulin Tyrosine Ligase (TTL), which is upregulated in paclitaxel-resistant tumors and promotes the retyrosination of tubulin, increasing septin binding to microtubules. The combination of those three events—high tubulin tyrosination, presence of polyglutamate chains, and septin recruitment—is necessary for efficient binding of Mitotic Centromere-Associated Kinesin (MCAK) and Cytoplasmic Linker Protein 170 (CLIP-170) to microtubules [93]. Those two proteins are a depolymerizing kinesin, and a rescue factor, respectively. They have been previously implied in taxane resistance [94,95] and their combination increases microtubule dynamic instability—a crucial property of taxane-resistant cells [96,97]. A follow-up study showed that upregulating septin isoforms present within octamers had a dominant effect on MTA resistance compared to overexpressing TTLL5 and TTLL11 [98], which confirms septins are a critical element of this mechanism of MTA resistance.

While the above-mentioned studies highlight the importance of SEPT9 in the context of septin–tubulin interactions, they do not explain SEPT9’s isoform specificity. In taxane-resistant cells treated with paclitaxel, septins are shifted from actin filaments to microtubules. This process is more pronounced in cells resistant to paclitaxel and is observed in cells expressing specifically i1 but not the i3 isoform of SEPT9 [98]. This suggests that septin octamers’ ability to bind microtubules depends on their i1 isoform levels. Since i3 and i1 isoforms differ only in the N-terminal 25 amino acids, this protein region is suspected to be responsible for this interaction. Indeed, the genomic analysis found two MAP-like patterns within this short sequence [57].

Interestingly, to bind tubulin successfully, MAP-like patterns must be repeated, which is executed by dimerizing SEPT9_i1 via the NC-NC interface and setting two N-terminal fragments nearby. This can be achieved either through direct dimerization [58] or through septin octamers—in which two SEPT9s occupy the central spot [9,98]. This implies SEPT9_i1’s dominant role in the etiology of taxane resistance and highlights the numerous pathological and physiological aspects of septin–microtubule interactions.

These findings solidify SEPT9_i1 as a potential target in the chemotherapy of treatment-resistant neoplasms. The newly described mechanism explains the results of the initial study [91] much better than previous theories, as it suggests that increased microtubule dynamic instability may confer resistance to both microtubule-stabilizing and microtubule-destabilizing drugs, as compared to competition with MAP4 [92,99]—a MAP known to counteract tubulin acetylation characteristic for paclitaxel treatment [100]. Altogether, further research focused on septin involvement in the development of drug resistance represents a promising direction.

**Figure 5:**
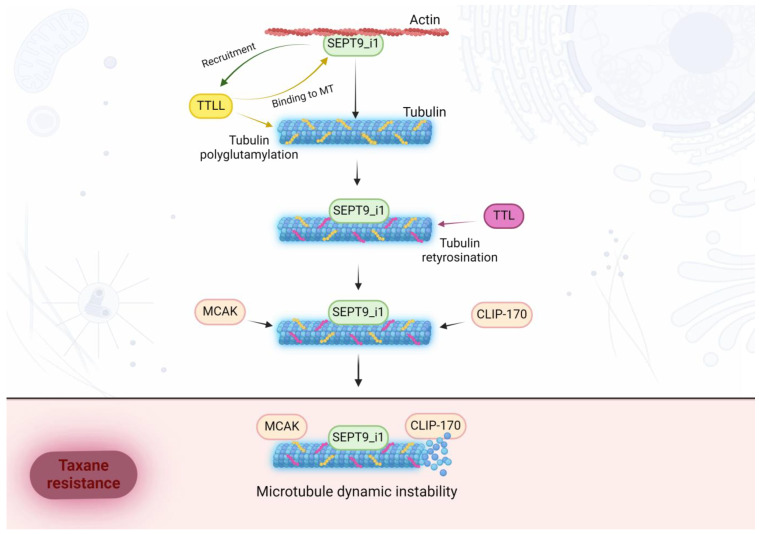
Schematic representation of molecular mechanisms leading to chemoresistance to taxanes. SEPT9_i1 (Septin 9 isoform 1) overexpression and relocalization to MTs (Microtubules) increase TTLL (Tubulin Tyrosine Ligase Like) recruitment, which results in tubulin long-chain polyglutamylation. TTL (Tubulin Tyrosine Ligase) is responsible for tubulin retyrosination. Both TTLL and TTL presence increase SEPT9_i1 binding to microtubules. Tubulin retyrosination and polyglutamylation, in turn, lead to higher CLIP-170 (Cytoplasmic Linker Protein 170) and MCAK (Mitotic Centromere-Associated Kinesin) recruitment to microtubules and enhance microtubule dynamic instability, which is a crucial property of taxanes resistant cells.

A schematic representation of the molecular mechanisms of SEPT9’s impact in shaping chemoresistance to taxanes is provided in Figure 5.

### 3.3. Competition and Synergism between SEPT9_i1 and Its Isoforms and Their Relevance to Cancer Biology

From the gathered evidence, SEPT9_i1 emerges as a scaffolding protein involved in the development of cancer cell motility and therapy resistance. Those tumor cells’ characteristics are greatly associated with poor clinical outcomes, pointing toward SEPT9_i1’s decent potential as a negative prognostic marker. However, SEPT9_i1 is not the only SEPT9 isoform investigated in the context of cancer biology. In the above-presented papers, SEPT9_i1 was most often compared to two other long SEPT9 isoforms—i2 and i3.

While SEPT9_i1 is the most promigratory septin, its expression alone does not translate to increased intravasation but solely increases metastatic capabilities after injecting tumor cells straight into the bloodstream [27]. Indeed, SEPT9_i3, not i1, has the greatest influence on cell’s ability to secrete matrix metalloproteinases (MMPs) and dissolve Extra Cellular Matrix (ECM) by facilitating MMP transport to FAs [32], suggesting complementary functions of SEPT9 isoforms. While i1 may promote migration and FA maturation, i3 promotes ECM degradation and FA turnover by degrading ECM particles that FAs are anchored to, limiting the mechanical tension responsible for the preservation of FAs. Other studies, however, reported a suppressive effect of SEPT9_i3 overexpression on cell growth, which may point to SEPT9_i3’s more ambiguous character [101].

SEPT9_i2, another long SEPT9 isoform, was shown to exhibit anti-migratory effects dependent on its unique N-terminus, the removal of which leads SEPT9_i2 to behave similarly to i1 and i3, and restores motility in SEPT9-depleted cells [102], suggesting its inhibitory effect on parts of the N-terminal domain upstream from this short, N-terminal sequence. According to certain studies, SEPT9_i2, opposite to other isoforms, was also shown to disrupt the formation of subnuclear stress fibers and stabilize actin polymers through myosin and cofilin activity inhibition [52]. Those findings line up with the most clinically explored direction of septin research—the examination of SEPT9_i2 promoter methylation as a potential biomarker for CRC [102,103]. Another suggested explanation is SEPT9_i2’s interaction with the WNT/β-catenin pathway [104]. Nevertheless, there is still not enough data regarding SEPT9_i2’s exact role in cancer development and suppression.

Out of the long isoforms of SEPT9, i1 is widely accepted as an oncogene, i2 is rather tumor-suppressive and i3 displays mixed properties. Due to the specific nature of septin interactions and their high homology, another mechanism explaining septins’ impact on cancer cell biology can be proposed. The oncogenic effects of i1 may be decreased by competition with other long isoforms for its protein partners. For example, while i3 can bind the HIF-1α particle via its G domain, it cannot facilitate its binding with importins [59,82], SEPT9_i3 can also replace one of the SEPT9_i1 particles within the septin octamer, disrupting the functionality of MBD [57]. This may explain the mixed properties of i3 compared to i1. While i3 has some oncogenic properties, its overexpression may disrupt critical pathways facilitated by i1. It may also contribute to the suppressive properties of i2. Understanding this interplay is crucial for a better understanding of septin dynamics and should be considered when comparing different septin isoforms.

## 4. SEPT9_i1 and Other Septins as Novel Therapeutic Targets in Cancer

### 4.1. Forchlorfenuron—Mechanism and Effects of Action

Forchlorfenuron (FCF)—a small-molecule synthetic plant cytokine widely used in agriculture has become a hallmark in septin research [105]. FCF was the first-discovered disruptor of septin dynamics and remains the most commonly used molecule in this application. FCF acts through binding the G domain and forcing conformational changes, which increase the formation rate of the large polymeric septin structures. This reduces the prevalence of more sophisticated septin heterooligomers and free-form septins, responsible for their cellular functions [106,107]. FCF treatment yielded promising results in vitro, limiting cell motility and proliferative capabilities in various cell lines, including colon, breast prostate cancer, and mesothelioma [108,109,110], as well as decreasing the expression of certain oncoproteins. The emerging role of septins in cancer cell biology and FCF’s role as a septin inhibitor has recently led to attempts at implementing septin inhibitors in cancer treatment. However, while FCF has promising effects on cancer cells both in vitro and in vivo, the high concentrations required for its efficacy limit its potential use as a chemotherapeutic [110]. The most promising direction seems to be searching for FCF analogs with a better affinity to specific oncogenic septin paralogs, such as SEPT9_i1 and SEPT9_i3. Some of such particles have already been developed, mainly by substituting or adding halogen groups to pyridyl moiety. Those FCF analogs were effective at causing a cytotoxic and antiproliferative effect on endometrial cancer [111] and mesothelioma cells [110,112]. The mesothelioma study showed that the effects of treatment with FCF analogs varied depending on the morphology of the used cell line—namely, their epithelioid/sarcomatoid phenotype. This may support the reasoning that finding inhibitors specific to septin paralogs overexpressed in tumors increases their therapeutic effect while minimizing toxicity. For now, however, the FCF analog’s in vitro effect seems to be directly correlated with its in vivo toxicity [112]. Furthermore, some off-target effects of FCF have been reported, which may warrant additional caution regarding adverse side effects in further search of clinically viable analogs [113]. Nevertheless, searching for FCF analogs that could constitute potential chemotherapeutics seems to be the most promising direction, even if it corresponds to fine-tuning the strength of the drug to achieve a suitable risk-to-benefit ratio. While not numerous, there are reports of other non-FCF derivative septin-targeting drugs, namely, Procyanidin B3 and 3013-0144. Procyanidin B3 has been proposed based on in silico analysis and is supposed to bind the G domain preventing septin dimerization, thus disrupting septin dynamics [114]. 3013-0144 was found via a yeast chemogenomics platform and shown to interact with Cdc12, a yeast septin. In a later comparison with FCF, 3013-0144 showed similar effectiveness in He-La cells at five times lower concentrations [115]. While, for now, those molecules are not as well examined and commonly used as FCF, they may prove helpful in future studies and therapies.

### 4.2. Septin Chemotherapy beyond Cytotoxicity

#### 4.2.1. Septin Inhibition as a Tool for Overriding Tumor Resistance to Other Forms of Treatment

Most of the presented studies approach septin inhibitors as potential cytotoxic drugs [111,112]. However, this is not the only possible approach. There is a plethora of data linking septin expression with a poor treatment response. Apart from SEPT9_i1, which has demonstrated the strongest association, SEPT9_i4 has also been found to contribute to taxane resistance [116]. Moreover, other studies have reported the involvement of SEPT10 in paclitaxel resistance [117], which could also be targeted by the administration of septin inhibitors.

Apart from taxanes, septin activity may also influence other chemotherapy treatments. For instance, SEPT2 was detected as a contributor to cisplatin resistance [118], and, as already mentioned, there are existing reports of SEPT9’s impact on MT-destabilizers efficacy on cancer cells [91,93,116]. SEPT9_i1 was also shown to have a pro-EMT function [101], which is considered to be a process highly connected with resistance to various forms of treatment. Another interesting report is SEPT9_i1’s protective effect on xenografts when exposed to radiotherapy [29]. Although the exact mechanism remains unknown, HIF-1 was implied as one of the major players in the development of radioresistance [119]. These reports suggest the potential application of septin inhibitors in various types of septin-overexpressing tumors, aiming to increase their effectiveness rather than to achieve cytotoxicity resulting from septin inhibition. The most obvious candidate seems to be breast cancer as it is commonly used in studies regarding SEPT9_i1 [27,28], and its treatment consists of a combination of chemotherapy, often involving taxanes, radiotherapy, and surgery [120]. Furthermore, there are reports of SEPT9’s involvement in stabilizing HER2 receptors [121], which are commonly targeted in breast cancer therapy [122]. Similarly, prostate cancer, which, in the case of aggressive tumors, is treated with radiotherapy and taxanes [123], might be an option worth considering. Another potential target for such treatment is ovarian cancer—a tumor with an initially high response to chemotherapy linked to the development of drug resistance later on [124]. A common drug combination used in the treatment of ovarian cancer is a taxane and platinum compound [125]. Moreover, SEPT9 overexpression was shown to be present in ovarian cancer cell lines [91], as well as in patient’s ovarian tumor samples [126]. A summary of the molecular interactions contributing to SEPT9_i1’s promotion of taxane resistance is presented in Table 1.

#### 4.2.2. Septin Inhibitors as Potential Migrastatics

Classical cytotoxic treatment primarily targets quickly multiplying cells. While this allows for sufficiently specific targeting of tumorous cells, it is not their only characteristic property. Of note, the ability to metastasize is perhaps the leading contributor to mortality in the case of solid tumors. This premise has led to the development of the term migrastatic, which describes a drug that targets specific cellular pathways and cytoskeleton regulation to decrease the risk of metastases and improve clinical outcomes [129].

Many, if not most, of the pathways reported in this review contribute to cell motility and invasiveness in one way or another. Both HIF-1 and JNK pathways have been linked to the formation of metastases [67,68,130,131]. Septin interactions with the actin cytoskeleton, especially involvement in the RhoA/ROCK pathway, are crucial to both mesenchymal and amoeboid types of migration and seem to line up with the current perspective on which part of cell machinery should be targeted in migrastatic therapy. The targeted mechanisms should be shared between the two aforementioned modes of migration, ensuring that tumor cells would not attain resistance by switching between the mesenchymal and the amoeboid type [129]. In vitro studies have already shown FCF’s ability to reduce cell motility, which might suggest utilizing this mechanism of septin disruption in migrastatic therapy [109,110]. Moreover, doses necessary for achieving mobility impairment are much lower than those required for cytotoxicity [132], which coupled with FCF’s relatively low toxicity [133], may enable clinical success with less pronounced side effects, often problematic in cancer treatment. Overall, while promising, there is still a long way before septin inhibitors will be considered viable drugs in cancer therapy. However, there is a strong theoretical basis to support further research in this direction (Table 2).

## 5. Conclusions

SEPT9_i1 emerges as a hub for various protein interactions crucial to cancerogenesis, metastasis, and treatment resistance, which points toward its potential as a negative prognostic marker. Due to the nature of septin dynamics, and high affinity toward numerous binding partners, these potential markers should always be analyzed within a specific context. The most important example of that is SEPT9_i1’s potential cooperation and competition with other long SEPT9 isoforms. However, based on current evidence, we can propose SEP9_i1 and septin dynamics as potential targets for cancer treatment. While the cytostatic potential of septin inhibitors has been the dominant approach to date, it is warranted to expand their potential implications in adjuvant therapy as migrastatics. Nonetheless, efforts should focus on improving the existing compounds’ properties and developing new septins for clinical use. Ultimately, septin research is a niche subject with its own set of unique challenges, warranting further exploration to unveil novel practical applications.

## Figures and Tables

**Table 1:** Proteins and interactions contributing to SEPT9_i1’s promotion of taxane resistance.

SEPT9_i1-Associated Protein	Mechanism of Interaction	Source
JNK, HIF-1 α	SEPT9_i1 increases HIF-1 α and JNK’s activity by preventing their degradation and, in the case of HIF-1 α, enhancing unclear shuttling [59,127].	HIF-1 α and JNK have both been implicated in the development of taxane resistance [68].
MAP4	SEPT9_i1 competes for the same binding spot on the microtubules with MAP4 [99].	MAP4 halts tubulin depolymerization—its inhibition counteracts the microtubule-stabilizing effect of taxanes [92].
TTL, TTLL5, TTLL11, CCP1, CLIP-170, MCAK	SEPT9_i1 scaffolds for TTLL5, TTLL11, and CCP1, which regulate tubulin polyglutamylation, increasing the recruitment of CLIP-170 and MCAK [93,98].	CLIP-170 and MCAK are crucial for maintaining microtubule dynamic instability—the state associated with resistance to MTAs [96,97].
-	SEPT9_i1 overexpression induces EMT in tumor cells in vitro—the exact mechanism and associated proteins remain unknown [101].	EMT is a process strongly implicated in tumor resistance to various forms of treatment, including taxane therapy [128].

**Table 2:** Proteins and interactions contributing to SEPT9_i1’s promotion of tumor cell motility and invasiveness.

SEPT9_i1-Associated Protein	Mechanism of Interaction	Source
ARHGAP4	SEPT9 and SEPT2 suppress ARHGAP4, which increases promigratory protein Rho activity [89].	RhoA/ROCK pathway is crucial to various cellular processes involved in tumor cell motility and its ability to metastasize, including stress fiber stabilization and the formation of FAs [134].
RhoA, ROCK	SEPT9_i1 increases activation of RhoA/ROCK pathway, leading to increased stress fiber formation and stability [27].	
FAK, Src, Paxilin	FA maturation is amplified through increased recruitment of FAK, Src, and paxillin facilitated by SEPT9_i1 [27].	Focal adhesions are involved in the formation of invadopodia, dissolution of ECM, and other processes involved in metastases [135].
JNK, HIF-1 α	JNK and HIF-1 alpha, known for their promigratory properties, are protected from degradation by SEPT9_i1 [59,127].	HIF-1 and JNK are known for their promigratory influence, with HIF-1 even being named the “master regulator of metastasis” [71,78].
Cortactin, TKS5	SEPT9_i1 recruits cortactin and TKS5 during the formation of juxtanuclear invadopodia. The exact mechanism remains unclear—stabilization of the nuclear envelope has been proposed as a contributor [88].	Invadopodia are some of the most important structures involved in tumor cell invasion and metastases [86].

## Data Availability

Data sharing is not applicable to this article.
